# Use of a Distal Radius Endoprosthesis Following Resection of a Bone Tumour: A Case Report

**DOI:** 10.1155/2009/938295

**Published:** 2010-03-02

**Authors:** Kishan Gokaraju, Kesavan Sri-Ram, James Donaldson, Michael T. R. Parratt, Gordon W. Blunn, Steve R. Cannon, Timothy W. R. Briggs

**Affiliations:** Sarcoma Unit, Royal National Orthopaedic Hospital (RNOH), Brockley Hill, Stanmore, Middlesex HA7 4LP, UK

## Abstract

Limited literature is available on the reconstruction of the distal radius using prosthetic replacement following resection of a bone tumour. We present the first reported case, in the English literature, of the use of an entirely metal endoprosthesis for the reconstruction of the distal radius. This case involves a 66-year-old male who was treated for giant cell tumour of the distal radius with surgical excision of the lesion and replacement of the defect using a predominantly titanium endoprosthesis. He was followed-up for 56 months following surgery and had a good functional outcome with no associated pain or complications. We propose that the use of a primarily titanium endoprosthesis for the reconstruction of a bone defect of the distal radius is a suitable alternative, providing good function of the forearm with satisfactory range of movement at the wrist and adequate pain relief.

## 1. Introduction

Developments in limb-salvage surgery have allowed the successful use of endoprosthetic replacements for the reconstruction of large bone defects following bone tumour resection. There are few reports, however, on the use of prosthetic implants for the reconstruction of distal radius defects. Previous methods of treatment following excision of distal radius tumours include arthrodesis of the wrist with autograft [1–6] and non-arthrodesed wrists using fibula autografts [7, 8] and osteoarticular allograft [9–11]. The literature on endoprosthetic replacement of the distal radius is minimal but has shown potential [12–14].

We present the case of a patient with symptomatic invasion of the distal radius by a juxta-articular giant cell tumour. This was treated with resection of the lesion and reconstruction of the resulting defect with the first ever custom-made, entirely metal distal radius endoprosthesis. Clinical and radiological evaluation of the patient was made to assess functional outcome and current status of the prosthesis.

## 2. Case

**Figure 1:**
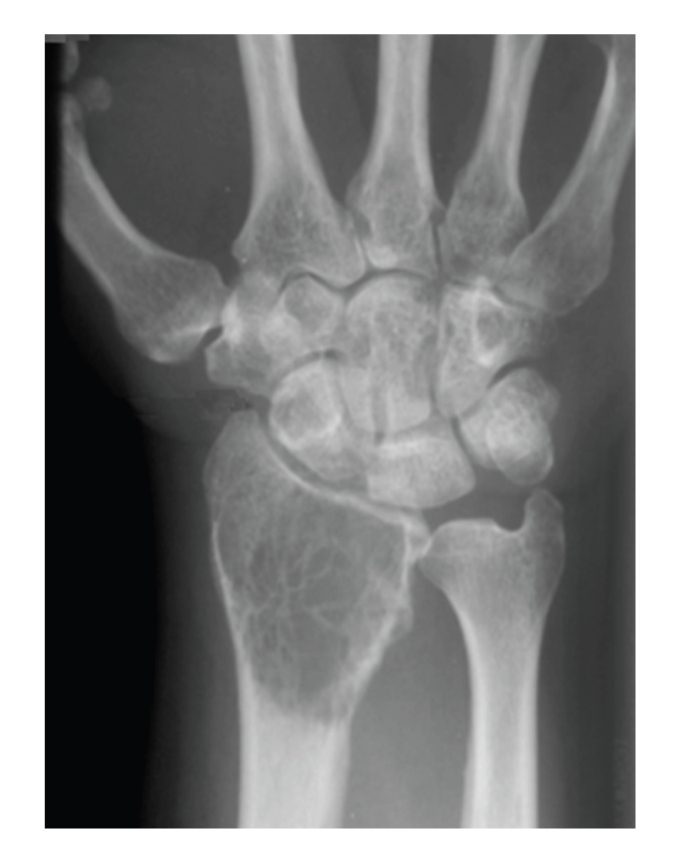
Preoperative radiographs demonstrating a giant cell tumour of the distal radius (seen as a classic lytic lesion).

A 66-year-old right-handed gentleman was referred for treatment of a giant cell tumour of the right distal radius. He had suffered with gradually worsening wrist pain and swelling for approximately six months without any previous history of trauma. Symptoms were worse at night and function was poor. He was otherwise fit and well with no other complaints. He had a colonic tumour resected eleven years ago and was clear of disease at his last check up. On examination, a firm, diffuse swelling was present on the dorsal aspect of the distal forearm and all movements of the right wrist were reduced secondary to pain. Wrist flexion and extension were limited to 10° each while radial deviation was absent and ulna deviation was reduced to 10°. Pronation and supination were reduced to 30° and 20°, respectively, from neutral. In comparison to the contralateral side, clinically, grip strength of the affected limb was significantly decreased. No neurovascular deficit was present. Radiographs of the forearm and wrist demonstrated a large lytic lesion of the distal radius, suggestive of a giant cell tumour of bone (Figure 1). This was confirmed following biopsy and a chest radiograph, whole body bone scan and MRI of the forearm revealed no other lesion.

**Figure 2:**
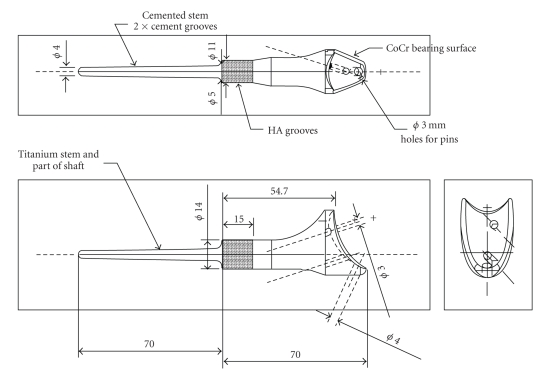
Template of a predominantly titanium distal radius prosthesis with a cobalt-chrome articulating surface (and measurements taken from contralateral forearm radiographs).

It was decided that, in view of the size of the lesion and the patient's age, resection of the tumour and subsequent reconstruction of the defect were to be performed using a distal radial endoprosthesis which articulated with the carpus. A custom-made predominantly titanium implant (Stanmore Implants Worldwide Ltd.) was constructed for use, which included a fixed cobalt-chrome articulating surface. Measurements for accurate manufacture of the implant were taken from radiographs of the contralateral forearm (Figure 2). It was essential that an anatomical fit was achieved to enable maximum function of the flexor and extensor tendons about the wrist joint. The implant included a tight-fitting proximal intramedullary stem and a hydroxyapatite(HA)-coated collar at the site of the bone-prosthesis interface to aid osseointegration. The requirement and design for this HA collar were based on previous the literature demonstrating both the growth of bone into and around the HA material, enhancing stability, as well as a decreased rate of aseptic loosening with massive endoprostheses [15, 16]. 

Via a dorsal incision, 70 mm of the distal radius, including the lesion, was excised along with the surrounding periosteum and the biopsy tract. It was confirmed that the cortex around the lesion was completely intact. The proximal radius was reamed and the prosthesis was inserted using gentamicin bone cement. Adequate reduction was ensured to provide maximum contact between the proximal hydroxyapatite collar and the distal cortex of the freshly cut bone. The stability of the wrist was then enhanced using the extensor carpi radialis longus, which was transected distally, passed through a premade design hole in the lateral section of the distal prosthesis and reattached to the capsule to form a lateral stabiliser. The capsule was then sutured down onto the soft tissue around the distal ulna, haemostasis was achieved and the incision was closed in layers. The limb was placed into an above-elbow plaster of Paris splint for four weeks. Post-operative radiographs suggested that the distal radius prosthesis was longer than intended despite the prosthesis sitting perfectly on the bone at the level of the resected radius.

At four weeks post-operatively the plaster of Paris splint was exchanged for a future splint. At six weeks, hand and wrist physiotherapy was initiated to start wrist flexion, extension, and rotation. The patient was followed up post-operatively with regular clinical and radiographic evaluation to assess symptom relief, hand and wrist function, and implant survival. At the most recent review, at 56 months, he achieved wrist dorsiflexion of 40°, palmar flexion of 20°, radial deviation of 10°, and ulna deviation of 20°. Elbow flexion, extension, and pronation were all full while supination reached 45°. On clinical assessment, the wrist was stable and the power of grip in the operated hand was full, equal to that of its contralateral counterpart. Hand and wrist function had improved significantly following surgery to give the patient satisfactory pain-free movement, allowing him to adequately carry out routine daily activities without difficulty. The patients functional score using the full DASH (Disabilities of the Arm, Shoulder and Hand) scoring system was 10.3 out of 100 [17]. A preoperative DASH score was unavailable for comparison but the intention of this study was not to make comparisons with preoperative function but to assess the outcome of the prosthesis with regards to function and survivorship.

**Figure 3:**
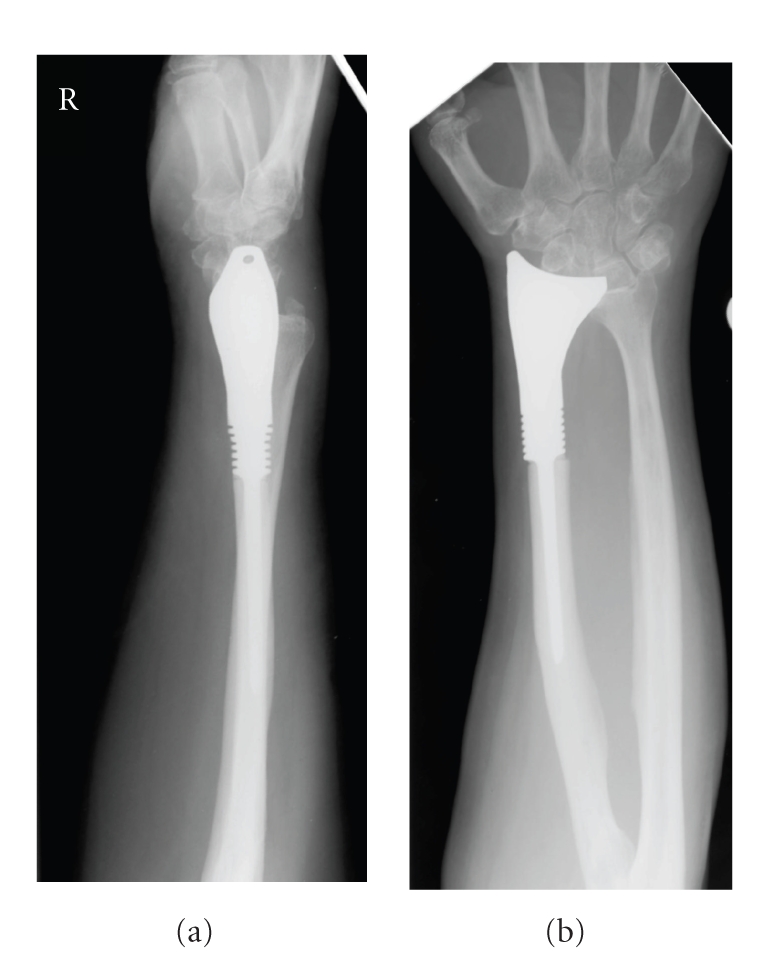
Radiographs of the distal forearm taken post-operatively at most recent follow-up, demonstrating the distal radial prosthesis and mild degenerative changes within the carpus.

The most recent radiographs demonstrated a secure fixation of the prosthesis without any signs of loosening around the intramedullary stem or recurrence of disease. The X-rays confirmed bone remodelling and osseointegration at the bone-prosthesis interface promoted by the proximal hydroxyapatite-coated collar. Due to the position of the distal articular surface of the prosthesis in comparison to the more proximal ulna styloid, there was some ulna translation of the carpus but this had only produced mild degenerative changes of the carpal bones, present along with age-related osteopenia (Figure 3).

## 3. Discussion

The primary aim of treatment of a giant cell tumour is to completely remove the tumour, avoid recurrence, and retain maximum possible function of the affected limb. The medium-term follow-up of our patient suggests a satisfactory outcome of the implant with regards to symptoms and function of the wrist and forearm. Range of motion at the wrist was reduced in all planes and supination of the forearm was decreased by 50%, most probably due to the increased length of the radius and disruption of the distal radio-ulna joint (DRUJ) following surgery. Unfortunately, this technique was not able to reconstruct the DRUJ. Despite this deficit in movement at the wrist and elbow, the patient managed competently with daily activities without pain or difficulty. There were no signs of loosening and there were no clinical or radiological signs of disease recurrence. The prosthesis did permit a degree of ulna drift of the carpus, resulting in mild degenerative changes within the carpal bones, but this also did not compromise the clinical outcome or survivorship. Future designs of articulating distal radius endoprostheses must consider such deficits and incorporate relevant changes. Greater accuracy would be required with regards to length of the prosthesis in order to prevent carpal subluxation. In addition, purpose-made holes in the DRUJ surface of the prostheses may be necessary for reconstruction of the DRUJ soft tissues, providing adequate stability to the joint.

Several articles have described recurrence of giant cell tumours following curettage of the primary lesion [1, 12, 14, 18–20]. Larger and more progressive lesions, particularly those which have penetrated the cortex and the periosteum, necessitate excision *en bloc *in order to minimise risk of recurrence [1, 11, 19, 21]. The use of autograft from various sites, with or without wrist arthrodesis, for the reconstruction of the resulting distal radial defect has been reported with varying success. Vascularised and nonvascularised iliac crest, proximal tibia, proximal fibula, and distal ulna grafts have been utilised to fuse the wrist joint following tumour resection [1, 2, 4–6, 22]. Alternatively, successful arthroplasty of the wrist joint allows preservation of wrist movement and this has been performed with proximal fibular autografts, cadaveric allografts, and prosthetic replacements [1, 7–14].

Distal radius reconstruction and radiocarpal fusion with autograft aims for secure union of the radius-graft and graft-carpus junctions, restricting movement at the wrist and elbow but yet maintaining satisfactory function [2–6, 8, 23]. Nevertheless, such operations can be prolonged, particularly with vascularised grafts, and complications including nonunion, graft or junctional fractures, and donor-site morbidity are well reported [1, 2, 5]. In addition, disruption of the extensor mechanism may occur from either the prominence of dorsal plates used for fixation or from resulting adhesions, thus restricting finger movement [1–4]. Seradge describes a method of ipsilateral distal ulna transposition across to the transected radius with fusion of the distal ulna end to the sacpho-lunate complex for the treatment of giant cell tumours. Successful fusion allowed 10 to 15° of flexion and extension at the intercarpal joint and 14-15 kg of grip strength allowing patients to resume with normal activities [6]. Griend discussed that this method of distal radius reconstruction does, however, leave a significant narrowing or “hourglass” appearance of the distal forearm that potentially may be aesthetically displeasing to some [1].

Minami et al. compared partial wrist arthrodesis to arthroplasty using vascularised fibula grafts and suggested that union of bone ends was equally successful in both but functional scores were greater with fibula-carpal fusion [8]. Griend et al. discussed that, despite preserving motion at the wrist, non-arthrodesed fibula grafting of the distal radius led to volar subluxation of the carpus in certain cases and degenerative changes [1]. This outcome was seen in our case with ulna deviation of the carpus but function appeared not to be significantly impaired. Carpal subluxation can be avoided in children by using a vascularised skeletally immature proximal fibula to reconstruct the defect following resection of distal radius tumours. It has been demonstrated that, following secure fixation of the graft, the transferred fibula epiphysis remodels over time from that of a flatter surface to a more concave articulation to accommodate the natural movement of the proximal carpal row. To ensure the success of this technique, it is imperative to ensure an adequate blood supply to the transferred graft via the accurately reanastamosed vasculature [24]. With all these methods of autograft reconstruction however, donor-site morbidity still remains an issue. This is absent with the use of osteoarticular allografts but this includes risk of infection and difficulty obtaining size-matched donors on demand. Kocher also observed volar dislocation of the carpus and graft failure with such allograft surgery, documenting an overall revision rate of approximately 21% (not including reoperations for recurrence). Arthroplasty using either allograft or autograft requires sound internal fixation and reconstruction of the radiocarpal ligaments for stability of the graft and the wrist joint, thus providing restoration of anatomy and mobility at the wrist [9, 10].

Endoprosthetic hemiarthroplasty of the wrist was described by Gold in 1957 for the treatment of recurrent giant cell tumours of the distal radius. Following initial treatments with curettage and then with fibular grafting, a decision was made to use an acrylic prosthesis with a stainless-steel intramedullary proximal stem, designed from radiographic measurements of the normal contralateral forearm. The concaved distal articular surface provided stability and enabled sufficient range of movement for the patient to resume normal activities within six months. At fifteen months postsurgery, flexion and extension of the wrist was 15 to 20°, pronation of the forearm was full, supination was 10°, and there were no restrictions in finger movement, still providing good function. At 21 months following surgery, the prosthesis fractured in the acrylic component just proximal to the wrist. The author suggested that if a similar prosthesis were to be made at the time of writing, perhaps Vitallium (an alloy containing 60% cobalt, 20% chromium, 5% molybdenum) should be used instead of acrylic [12]. More recently, Hatano et al. discussed the long-term follow-up of two patients who each had a cemented alumina ceramic prosthesis for the reconstruction of the distal radius following tumour excision. Both patients described no pain and demonstrated good functional outcomes despite a gradual decrease in the radiocarpal joint space which resulted in ulnacarpal abutment and significant radiographic degenerative changes [14].

Use of a cemented titanium endoprosthetic distal radius replacement for the primary treatment of a giant cell tumour was used in this case. At medium-term follow-up, there was no recurrence or implant failure, and the technique provided good functional results, despite a decreased range of movement at the wrist and elbow. It allowed the patient to get back to work and resume daily routines without pain or discomfort. Clinically and radiographically, the prosthesis has not demonstrated any loosening and only mild signs of degenerative changes within the carpus. Wrist hemiarthroplasty using a titanium endoprosthesis is an option for reconstruction of the distal radius following giant cell tumour of bone. Long-term follow-up of this method is needed.
